# Systemic and organizational barriers to primary chronic disease prevention: a qualitative study of public health organizations in Canada

**DOI:** 10.24095/hpcdp.46.5.01

**Published:** 2026-05

**Authors:** Katerina Maximova, Maryam Marashi, Erin K. O’Loughlin, Jennifer L. O’Loughlin

**Affiliations:** 1 MAP Centre for Urban Health Solutions, Li Ka Shing Knowledge Institute, St. Michael's Hospital, Toronto, Ontario, Canada; 2 Dalla Lana School of Public Health, University of Toronto, Toronto, Ontario, Canada; 3 Faculty of Kinesiology and Physical Education, University of Toronto, Toronto, Ontario, Canada; 4 CHUM Research Centre (CRCHUM), Montral, Quebec, Canada; 5 cole de sant publique de l’Universit de Montral, Montral, Quebec, Canada

**Keywords:** chronic disease prevention, public health organization, organizational capacity, barrier, Canada, qualitative

## Abstract

**Introduction:**

Public health organizations in Canada play a central role in chronic disease prevention (CDP) but face persistent challenges, including system restructuring, persistent underfunding and shifting policy priorities. The growing complexity of these issues warrants qualitative insight to complement quantitative reports capturing CDP organizations’ perspectives.

**Methods:**

The Public Health Organizational Capacity Study (PHORCAST) is a repeat Canada-wide census of public health organizations engaged in primary CDP at national, provincial, territorial and regional population levels. In 2023, senior managers and staff with in-depth knowledge of their organizations’ CDP activities completed a questionnaire that requested optional comments via an open-ended question. The responses were analyzed using qualitative descriptive methods and inductive content analysis to identify and organize recurring issues. Theme frequencies are reported descriptively to indicate prominence across organizations and not to quantify meaning.

**Results:**

Across the 55 organizations, 125 coded references to barriers to CDP were synthesized into five key themes: organizational capacity and program delivery challenges (n=38), including chronic underfunding, workforce shortages and limited infrastructure; COVID-19 pandemic disruptions causing staff redeployment and prolonged service interruptions (n = 30); policy and systemic barriers (n=28), including political interference and poor interjurisdictional coordination; fragile partnerships and the need for stronger intersectoral collaboration (n = 16); and difficulties engaging diverse communities, digital access issues and lack of culturally responsive programming (n = 13).

**Conclusion:**

CDP efforts in Canada are constrained by structural, operational and contextual barriers. Addressing these challenges requires sustained investment, coherent policies and stronger cross-sector partnerships.

**Highlights**

- Chronic underfunding and workforce shortages are major barriers to primary chronic disease prevention (CDP) across Canada.
- Policy fragmentation, political interference and weak interjurisdictional coordination continue to undermine long-term CDP capacity.
- The COVID-19 pandemic intensified existing challenges through staff redeployment and disruptions to CDP programs.
- Reaching diverse communities is hindered by digital inequities and a lack of culturally responsive approaches.
- Partnerships are essential but remain fragile, which emphasizes the need for more stable, crosssector collaboration frameworks.

## Introduction

Chronic, non-communicable diseases, including cardiovascular disease, cancer, diabetes and chronic respiratory diseases, are leading causes of morbidity and mortality, globally. Public health organizations in Canada and elsewhere play a central role in developing and delivering chronic disease prevention (CDP) programs, policies and practices to reduce the chronic disease burden. Despite strong evidence supporting the cost-effectiveness of prevention, CDP remains underresourced and underprioritized within public health systems.^1^-^6^ Acute care continues to dominate policy and funding agendas, limiting investment in prevention.^7^-^10^ Short-term project-based funding contributes to policy instability, workforce turnover and loss of institutional memory, hindering the sustainability and scalability of evidence-informed CDP.^7^-^11^ Further, the COVID-19 pandemic exacerbated these challenges through staff redeployment and prolonged disruptions to preventive services, particularly in marginalized populations.^12^-^14^

In Canada, CDP is delivered by many different organizations mandated to support population-level primary prevention and health promotion, including formally mandated government organizations such as public health units or agencies, health authorities, and non-governmental organizations such as health charities and not-for-profit organizations.^1^-^6^ Collectively, these organizations operate within a public health system affected by decades of restructuring, fiscal constraint and shifting political priorities.

To monitor CDP capacity within this ever-evolving context, the Public Health Organizational Capacity Study (PHORCAST; https://www.celphie.ca/phorcast) was launched in 2004 as a recurring Canada-wide census of public health organizations that develop and/or deliver CDP initiatives at national, provincial, territorial and regional levels.^1^,^2^,^15^-^20^ All PHORCAST organizations are mandated to conduct population-level primary CDP and/or healthy lifestyle promotion. While PHORCAST has provided valuable quantitative data on organizational capacity measures,^1^,^2^,^16^ interorganizational collaboration and resource networks^15^ and trends in public health strategies for CDP and healthy lifestyle promotion,^17^-^20^ less is known about how public health organizations experience systemic barriers to CDP. 

In this qualitative sub-study of CDP organizations, we draw on PHORCAST data collected in 2023 to explore perceived pressures, including chronic underfunding, policy shifts, leadership transitions, staff redeployment and structural reforms. Drawing on the strengths of qualitative inquiry to illuminate organizational experiences and adaptations,^21^,^22^ we aim to provide a contextualized understanding of the operational realities shaping CDP in Canada today.

## Methods

A qualitative sub-study was embedded within PHORCAST 2023, which collects data from all national, provincial, territorial and regional public health organizations with mandates for primary CDP in Canada.^1^,^2^,^16^,^17^,^19^ Organizations that are eligible to participate in PHORCAST include provincial, territorial and regional health authorities, public health units and agencies, government departments, para-governmental agencies (i.e. those financed by government but acting independently), national health charities and their provincial, territorial or regional chapters, other non-governmental and not-for-profit organizations, resource centres and professional associations. The inventory of organizations was compiled in 2004 and updated in 2010 and 2023. 

We first generated an exhaustive list of candidate organizations in each province and territory and nationally, through rigorous Internet searches. We then validated the list to ensure completeness by consulting with experts with wide-ranging knowledge of the public health landscape at the national, provincial, territorial or regional levels in Canada. To update the inventory in 2010 and 2023, we confirmed the continued existence of organizations that did or did not participate in the previous data collection waves, including previously ineligible organizations, because organizational mandates can change over time. We then conducted further Internet searches and consulted other sources (i.e. provincial and territorial mailing lists, membership databases, experts in each province and territory as well as national experts) to identify organizations that began operations since the preceding census and confirmed their eligibility according to their mandate. We also confirmed the eligibility of existing organizations functioning with new CDP divisions or offering new types of activities; and those formed by the amalgamation of two or more previously participating organizations.

The inventory included public health organizations that develop or adapt primary CDP initiatives (e.g. programs, policies, practices) and transfer these initiatives to other organizations (referred to as “resource organizations”); and/or deliver or implement CDP initiatives for the population-at-large or specific subgroups (referred to as “user organizations”). Specifically, included were public health organizations with mandates for population-level primary prevention of chronic disease (cancer, cardiovascular disease, diabetes, chronic respiratory diseases) or healthy lifestyle promotion, or with a single-focus mandate for healthy eating, tobacco control or physical activity. Organizations that focused exclusively on secondary or tertiary prevention, research, fundraising, advocacy or knowledge translation were excluded as were those that operated only at the local level.^1^,^2^,^15^-^20^

In 2023, all eligible organizations (n = 335) were invited to participate. A senior manager from each organization was first contacted to confirm eligibility and to identify a key informant—defined as the individual most knowledgeable about the organization’s CDP activities. Senior managers could nominate themselves or another staff member. The key informants were contacted by email to confirm their suitability and subsequently invited to complete the PHORCAST questionnaire, which was available online on the LimeSurvey platform (LimeSurvey GmbH, Hamburg, DE). They could complete the questionnaire independently or be interviewed by the study coordinator or an investigator via Zoom (Zoom Communications, San Jose, CA, US), in accordance with standard PHORCAST procedures.^23^,^24^

The key informants (henceforth referred to as “participants”) were instructed to respond on behalf of their organization, reflecting collective experiences rather than their individual perspectives. Following closed-ended questions, the participants were invited to provide additional comments with a broad, non-directive, open-ended question: “Do you have any additional comments?” 

This sub-study is based on written or verbal responses pertaining to barriers to CDP from 70 organizations. Of these, 15 were excluded because they focused solely on questionnaire feedback, which yielded an analytic sample of 55 participants.

We adopted a qualitative descriptive design to summarize organizational perspectives on barriers to CDP with minimal interpretive inference.^25^,^26^ This approach was selected to provide a practice-relevant description of reported experiences that used the participants’ language. Data were analyzed using an inductive content analytic approach, with codes derived directly from the data rather than imposed a priori. Two researchers independently coded all responses and developed a shared codebook through iterative discussion. Minor differences (12 of 125 coded references; 9.6%), typically reflecting the identification of an additional relevant category by one of the coders, were resolved through discussion.

Of the 55 organizational responses, 29 were provided verbally via Zoom interviews and 26 were submitted in writing. Verbal responses were generally more detailed (median of 236 words; range of 76–1604 words), whereas written responses tended to be shorter but substantive (median of 58 words; range of 9–178 words). Each response could be coded to more than one category. The frequency with which categories appeared across responses was tallied; this is reported descriptively to convey the distribution of reported issues across organizations. 

Pattern identification was informed by the general principles of thematic analysis described by Braun and Clarke, particularly the identification of recurring meanings across a dataset through recursive engagement with the data, without adopting reflexive thematic analysis as a standalone methodological framework.^27^ Analytic credibility was supported through independent dual coding, iterative team discussions and cross-checking of coding decisions made by researchers with different disciplinary and experiential backgrounds. We used reflexive discussions to support analytic transparency by considering how researchers’ professional experiences in public health and system-level initiatives may have shaped analytic decisions, consistent with qualitative descriptive practice. 

All transcripts were anonymized. Participant quotations are identified using the uppercase letter P and the anonymized participant number (e.g. *P* 50).

***Ethics approval***

Study procedures were approved by the Unity Health Toronto (21-240) Research Ethics Board and the CHUM Research Centre (2022-10366) Research Ethics Board. Informed consent was obtained from all participants.

## Results

In 2023, PHORCAST surveyed 298 public health organizations with mandates for CDP, which represented 89% of those eligible. The median age of the 55 organizations that provided responses to the open-ended question was 50 years. Most (75%) were user organizations and 47% were formally mandated government organizations. Over half of the organizations (58%) were entirely dedicated to CDP and 42% housed CDP units. Organizations served diverse geographic areas, with the largest proportion operating at the regional (31%) or provincial or territorial levels (40%). About half (51%) served geographical areas with more than 500 000 inhabitants (Table 1).

**Table 1:** Characteristics of CDP organizations that provided comments about barriers to CDP (n = 55), PHORCAST, Canada, 2023

Characteristics	Proportion
Median age, years (IQR)	50 (25–100)
User organization, %	75
Resource organization, %	15
FMO, %	47
NGO, %	53
Organizations entirely dedicated to CDP, %	58
Organizations housing CDP units, %	42
Geographic area served, %	
Subregion	7
Region	31
Province/territory	40
Multi-province/territory	11
Canada	11
Population size served, %	
< 50 000	7
50 000–99 999	2
100 000–199 999	27
200 000–499 999	13
500 000–1 000 000	13
> 1 000 000	38
Median number of full-time CDP staff, n (IQR)	67 (8–300)
Median number of volunteers, n (IQR)	25 (9–60)

**Abbreviations: **CDP, chronic disease prevention; IQR, interquartile range; FMO, formally mandated organization; NGO, nongovernmental organization; PHORCAST, Public Health Organizational Capacity Study. 

**Note: **Organizations categorized as “multi-province/territory” and “Canada” are distinct and do not overlap. 

Participants described multiple and overlapping barriers shaping CDP activities at the organizational level. Across the 55 organizations, 125 coded references were identified and subsequently synthesized into five key themes (Table 2). The most frequently mentioned barriers related to organizational capacity and program delivery (n=38); COVID-19 pandemic disruptions of CDP activities (n=30); and policies and systems (n=28). Less frequently mentioned were barriers related to building collaborations and partnerships (n=16) and engaging diverse communities (n=13) (Table 2).

**Table 2:** Themes in participants’ descriptions of organizational barriers to CDP, PHORCAST, Canada, 2023

Key theme	Frequency, n
Organizational capacity and program delivery	38
COVID-19 pandemic impacts on CDP activities	30
Policies and systems	28
Collaboration and partnerships	16
Reaching and engaging diverse communities	13

**Abbreviations:** CDP, chronic disease prevention; PHORCAST, Public Health Organizational Capacity Study. 

**Note:** A total of 125 coded references to barriers to CDP were identified across 55 organizations and synthesized into 5 key themes. The median number of themes mentioned by participants was 2, with a range of 1–5. 

***Organizational capacity and program delivery***

Chronic underresourcing emerged as a central challenge. Participants described a chronic state of “doing more with less,” exacerbated by inflation and flat budgets. Budget allocations often failed to reflect the growing scale and complexity of CDP work. One participant explained that despite rapid program growth:

… we’re still only getting the same money we’ve always gotten and there’s no accommodation for cost of, you know, the consumer price index, in terms of things going up, you know, our consulting fees, that type of thing are going up [P 50, resource organization]. 

This financial stagnation created practical obstacles across all levels of implementation, particularly for smaller and more remote units. Some organizations described how geographic isolation complicated their ability to attract talent and secure adequate resources:

We’re a very small organization in our province, we are smallest, by just 35000 people. And so it’s always hard for us to get those resources. And we’re also very, very far from most geographic centres so it’s hard to get students that are willing to come or move or participate in that way [P 118, user organization]. 

Meanwhile, the demand for preventive services (e.g. community-based lifestyle programs, school and workplace health initiatives, chronic disease screening, public education or awareness campaigns) has rebounded considerably since pandemic restrictions eased, placing additional strain on limited personnel and infrastructure. Many organizations reported that their capacity to meet this demand has not kept pace: “As things opened up, the demand has been high for CDP, but training and resourcing of critical programs has been low” [P 165, user organization].

The result is a responsive workforce that is stretched to its limits, striving to meet rising community needs, but lacking the financial and human resources necessary to sustain existing programs, let alone innovate or scale new ones. Several organizations expressed frustration that, despite good ideas and intentions, structural constraints limited their ability to pursue ambitious or high-impact programming:

There’s so much when you’re in a resource-constrained environment… you can be building the best program you can, but then you don’t always have the funding to execute it at scale and spread it. We always have to make choices about where we can reach.… [P 197, user organization]. 

Another participant similarly reflected:

We’ve had to do our budgets so that we don’t have any money in there for training our activity coaches any more because we need to survive as an organization—but we have to go out and get other funding and more money to implement the activity coaching [P 50, resource organization]. 

These perspectives illustrate how limited funding not only impedes day-to-day operations but actively diverts organizations away from proactive capacity-building or long-term planning. As one participant put it, “we’re just not resourced for that level of implementation” [P 197, user organization].

In short, while innovation and scale are aspirational goals, organizations remain entrenched in survival mode, making strategic trade-offs that prioritize feasibility over more ambitious programming.

***Policy and systemic barriers***

Participants depicted a policy landscape that undervalues prevention and forces organizations to operate reactively rather than strategically. The funding environment was described as volatile, often driven by short-term political agendas rather than long-term health outcomes. This instability compels organizations to continually adjust their priorities to secure funding, a practice that undermines continuity and strategic planning:

… if the federal government or provincial government have priority and then that’s where you’re going and then in the next year, they change the priority and you’re chasing that money, you don’t ever get momentum like you need because you’re changing lifestyle[s]. It takes years…. You can’t do it in a six-week program. It just doesn’t happen [P 144, user organization]. 

This short-termism was echoed by others who described a fragmented policy environment that lacked overarching vision or coordination. Instead of integrated, evidence-based strategies, organizations often encounter a scattershot approach at the provincial or territorial level:

What they’ve done is a patchwork of one-offs, as opposed to a comprehensive approach to this work.... I think there’s significant opportunity for us in [Province Name], if the ministry and the provincial government were participatory in looking at some of these issues and bringing [the] resources that they have [P 57, user organization]. 

Participants also emphasized that political and organizational cultures tend to prioritize visible, easily quantifiable programs over upstream policy measures that promote population health. This focus on short-term optics undermines investment in longer-term prevention strategies:

The lack of understanding, but also ... politicians are very focused on programming to say, you know, they want to be able to count numbers and so they don’t have the same recognition or understanding of how important healthy public policy is to creating a healthier population [P 185, user organization]. 

Similarly, frustration was expressed that despite consistent calls to rebalance funding from acute care toward prevention, health promotion continues to receive insufficient and only short-term investment:

The lack of recognition that health promotion will pay off, will give dividends into the future ... health care cost-saving initiatives target health promotion activities in the short term, which impact the long-term health and wellness of individuals.... Even though there’s been numerous reports about the shifting of funding from acute care to health promotion and population health, that has yet to take place [P 118, user organization]. 

In addition to funding and policy gaps, broader structural constraints were highlighted. Public health units embedded within government structures often face limitations in their ability to communicate directly with the public, particularly around sensitive or politicized issues. This restriction limits the visibility of prevention efforts and hampers timely public health messaging:

There’s a lot of political interference in public health because it’s not an independent office … but it makes it really difficult because … we do a lot of work in communications, social media, campaigns [and] all these sorts of things, developing fact sheets and infographics that would be helpful in knowledge transfer to the public. But they don’t get released, because they get stopped by somebody at a senior level [P 185, user organization]. 

Such interference reinforces the long-standing imbalance between acute care and prevention, a theme raised repeatedly. Several participants noted that, despite chronic diseases accounting for the majority of morbidity and mortality, prevention efforts continue to be overshadowed by reactive care priorities: “I think CDP is hugely underfunded…. We lose more people to chronic diseases than to communicable diseases.…” [P 135, user organization].

In addition to political interference, participants described sector-specific structural challenges that hindered organizations’ ability to deliver community-based prevention programming. One such issue was the loss of volunteers following the COVID-19 pandemic, a workforce that historically played a vital role in program delivery. As one participant explained:

Volunteers have not returned. So whether it’s [because] they don’t want to get sick or whether it’s [because] they had 18 months to 2 years of not volunteering, and they thought, like, this is great, I don’t want to volunteer anymore…. We’re trying to tease out exactly what’s occurring at a community level [P 142, user organization]. 

Together, these accounts paint a picture of a system that is continually reset by shifting political winds and hampered by institutional barriers to communication and continuity. The cumulative effect is a CDP sector unable to realize its full potential due to inconsistent investment, insufficient autonomy and a policy environment that often prioritizes visibility and short-term gains over sustained population health improvements.

***The impact of the COVID-19 pandemic on CDP activities***

Participants generally described the COVID-19 pandemic as a profound disruption to CDP efforts, with impacts reverberating far beyond the acute crisis period. As public health systems pivoted toward emergency response, almost all CDP programs were paused, defunded or sidelined. Many organizations had their mandates temporarily suspended as staff were reassigned to outbreak management and vaccination efforts. As one participant explained, “From March 2020 to about, I would say, about March 2021, we really didn’t do any chronic disease prevention work at all” [P 163, user organization]. Others described a near-total cessation of long-standing health promotion activities, resulting in a disconnect between organizations and the communities they serve; for example, “… a complete halt in all our Healthy Communities activities … [which left us] … at a disadvantage in understanding what has happened in our communities over the last number of years” [P 149, user organization].

In addition to halting service delivery, the COVID-19 pandemic caused cascading delays in program implementation, research and evaluation. The ripple effects of this disruption were felt across timelines, partnerships and strategic planning: “COVID-19 delayed and slowed timelines around some of the key transfer activities … it probably set teams back … 2 years” [P 79, resource organization].

Beyond operational setbacks, participants described lasting damage to internal cohesion and external collaboration. The redeployment of staff fragmented teams, diverted attention from core mandates and weakened organizational culture. Long-standing community partnerships were also strained or entirely lost: “The partnerships in the community have been affected. The relationships within the division … have been impacted … structural system changes … created barriers” [P 185, user organization]. These relational losses were compounded by a reluctance to return to in-person activities. While some organizations attempted to resume group-based programs, they encountered reduced participation and persistent fear: “The number one thing that we’ve seen … is the lack of people that want to come back after … because of the overall fear of being in groups … there isn’t like a set solution for it” [P 175, user organization].

Although a few participants identified silver linings such as an increased awareness of the social determinants of health or the importance of stronger community ties during crises, most conveyed a sense of a sector still in recovery. For many, the transition back to routine CDP work remained partial or symbolic, constrained by unresolved bottlenecks and ongoing pandemic-related priorities: 

After a year, people agreed that … it’s time to pull health promotion and let them get back to their own business, but it didn’t really mean anything because we couldn’t get anything released. It was still COVID-19 [P 185, user organization]. 

These reflections illustrate how the pandemic not only interrupted CDP activities in the short term, but also introduced additional systemic and relational barriers. The cumulative impact includes fractured teams, stalled innovations, weakened partnerships and lingering uncertainty about the future of prevention within public health systems still shaped by crisis response.

***Reaching and engaging diverse communities***

Participants highlighted ongoing barriers to ensuring that CDP efforts reach and resonate with diverse communities equitably. Many reported that standard models of program delivery—especially those that rely heavily on digital platforms—risk excluding populations already underserved by the health system. While digital tools expanded reach for some, they introduced new barriers for others. As one participant observed, “We focus on racialized or marginalized communities, in particular Indigenous Peoples … a virtual world doesn’t resonate with their cultural preferences” [P 69, user organization]. This insight reflects a broader recognition that technological solutions, while efficient, are not culturally neutral. For many communities, particularly those with distinct worldviews or histories of marginalization, digital engagement may feel disconnected or inaccessible.

Digital exclusion was also flagged as a major issue for older adults, who are often less comfortable or equipped to navigate online systems. Participants described the challenge of balancing innovation with accessibility, especially in rural or underserved areas with limited Internet infrastructure:

We’re still dealing with the 55‑plus, 50‑plus group—[they] have to use all mediums, including print and snail mail. A lot of things are only available online, [which is] not helpful, especially when parts of our province don’t even have Internet … there are 87‑year‑olds who never had a computer and don’t want one. Reaching out to certain people within a demographic … it’s not a homogeneous group [P 172, user organization]. 

These digital and generational divides were compounded by rising economic pressures, which further limited access to programs and supports. One participant emphasized how financial strain—especially for people on fixed incomes—has deepened pre-existing inequities: “With rising costs of things, it’s gotten worse in terms of accessibility for certain people … on fixed incomes … we see a lot of people having challenges now that they didn’t before” [P 172, user organization].

Participants called for a shift away from narrow, individual-level interventions, and toward models that are more holistic, inclusive and community-driven. There was a clear push to embed equity, cultural safety and Indigenous knowledge systems into CDP practice. One organization described its evolving approach:

We focus on community as client, not individuals, and are moving away from modifiable risk factors to equity, racial equity, built environment, etc. We’re learning and growing … trying to be humble and open to Two‑Eyed Seeing and new ways of knowing [P 61, user organization]. 

These accounts point to a growing awareness that achieving equitable CDP outcomes requires more than adaptation; it requires transformation. Programs must move beyond generic risk messaging and embrace delivery models that reflect cultural values, address economic realities and are co-designed with those they aim to serve. The call is not just for inclusion but for meaningful partnership, grounded in respect, reciprocity and relevance.

***Collaboration and partnerships***

Participants consistently emphasized that collaboration is both essential and challenging within the current CDP environment. While cross-sector partnerships are widely recognized as key to addressing complex determinants of health, many described a fragmented system where collaboration is encouraged rhetorically but unsupported structurally. Organizations often find themselves competing for limited funds, which undermines trust and shared action. According to one participant:

It seems like everyone is singing the same song, but from different parts … we’re all vying for the same grants and the same funding … we meet these organizations, and we say, how can we help each other, but then there’s no funding to increase the capacity for organizations to grow together [P 95, resource organization]. 

In the absence of sustained funding and infrastructure to support partnership work, collaboration often remains informal, short-term or dependent on personal relationships rather than system-level design. The same participant proposed a model where funders would incentivize and coordinate joint efforts: “We’ll give you a grant if you can play well together … and we'll have an intermediary placed with you, to help you…” [P 95, resource organization]. This vision suggests the need for intermediary structures—such as backbone organizations, neutral facilitators or conveners—that can bridge gaps between sectors and reduce the administrative burden on overextended CDP teams.

Others echoed the call for system-level coordination and saw a key role for provincial or territorial leadership in aligning local initiatives. Rather than massive new investments, participants advocated for smarter governance and facilitative leadership that could reduce duplication and increase collective impact:

If the ministry and if the provincial government were participatory … and what I mean by that is not significant amounts of further investment…. I get the sense that people will move in a variety of directions, which creates a very chaotic environment [P 57, user organization]. 

The pandemic also brought into sharper focus the vital role of community-based relationships in responding to public health needs. In some cases, the crisis acted as a catalyst for deeper engagement and cooperation around the social determinants of health: 

[The COVID-19 pandemic] actually became an enabler, particularly around work that’s happening in settings organized around social determinants of health. To be able to have broader stakeholders identify and recognize the need for action around social determinants of health [P 79, resource organization]. 

Several participants identified promising innovations, such as physician-linked activity coaching, that could benefit from stronger cross-sector infrastructure and investment. These models have the potential to bridge clinical and community care, but only if they are better integrated into broader systems of support:

We need more resources to do our thing … activity coaching is really going to be a game changer … We’re just sort of baffled as to why isn’t there more out there in terms of support for, you know, what we do and having physicians and allied health professionals prescribing [P 143, user organization]. 

Overall, these reflections suggest that partnerships are lacking in the scaffolding required to make them sustainable and effective rather than in willingness to collaborate. Modest but strategic investments in coordination, shared infrastructure and policy alignment could enable a shift from ad hoc collaboration to more durable and influential collective CDP.

## Discussion

This qualitative analysis extends earlier PHORCAST waves by highlighting what is newly salient in 2023, not simply what persists. Participants described the compounding effects of long-standing underfunding alongside post-pandemic inflation, workforce attrition and weakened partnership infrastructures, resulting in a qualitatively different form of capacity strain. The COVID-19 pandemic was consistently framed as an amplifier of pre-existing structural vulnerabilities, rather than a singular explanatory factor, accelerating processes already underway. Compared with earlier PHORCAST findings,^1^,^2^,^15^-^20^ participants’ accounts suggest that the erosion of CDP capacity appears more entrenched and less recoverable, with fewer organizational buffers available to absorb system shocks. The continued reliance on short-term funding cycles, combined with rising expectations for equity-oriented and community-responsive programming, places additional and novel strains on organizations operating in an increasingly volatile policy and fiscal environment.

A prevailing theme across responses was the unsustainable expectation for public health organizations to “do more with less,” a situation exacerbated by inflation and stagnant budgets. These constraints not only limit the delivery of CDP initiatives but also diminish capacity for innovation, evaluation and scale-up. These concerns echo earlier critiques of the inadequate prioritization of health promotion within Canadian health policy frameworks.^3^-^8^ As organizations are forced to triage basic service delivery over strategic development, the transformative potential of prevention is significantly undermined.

Policy instability and fragmentation emerged as additional threats to CDP capacity. Participants described an unpredictable funding environment that compels organizations to “chase the money” in response to shifting political priorities, precluding long-term planning and sustained momentum. This aligns with broader criticisms of Canada’s fragmented public health governance and its consequences for system integration and health equity.^3^-^8^ Without a stable and coordinated strategy for CDP at the provincial, territorial and federal level, efforts remain siloed and duplicative and are often limited to short-term initiatives that lack coherence.^28^

The COVID-19 pandemic further destabilized CDP operations, exposing long-standing vulnerabilities in public health infrastructure. Most organizations experienced widespread staff redeployment and program suspensions, with residual effects extending well beyond the acute phase of the crisis. These findings reflect global analyses of the pandemic’s disruptive effects on prevention and health promotion sectors.^12^ In addition to operational setbacks, participants reported long-term impacts on internal team cohesion and external partnerships—underscoring that CDP recovery must involve not just restarting programs but rebuilding organizational and relational foundations. These lessons also extend beyond pandemic recovery, highlighting the need for resilient CDP systems capable of withstanding other potential system disruptions such as climate-related emergencies (e.g. wildfires), economic downturns or digital infrastructure failures. Strengthening organizational flexibility and intersectoral coordination is essential to ensuring that prevention systems can adapt and continue to function in crises.

Equity-related barriers were also prominently described across organizations. While digital delivery models expanded during the pandemic, they often failed to engage older adults, low-income populations and culturally diverse communities. The participants emphasized the need for multimodal, culturally grounded approaches that go beyond individual risk messaging to address the structural determinants of health. This reflects a broader shift in public health discourse toward equity-centred and community-based frameworks.^29^-^31^ Several participants called for the integration of Indigenous knowledge systems, such as Two-Eyed Seeing, highlighting the potential of reconciliation-informed models of CDP that honour diverse ways of knowing.

While collaboration was seen as essential for effective CDP, participants described it as difficult to sustain. They noted that current funding and governance arrangements offer little support for sustained partnership work. Previous research suggests that long-term success requires alignment across systems, supportive infrastructure and mechanisms for coordination.^32^ Suggestions for intermediary supports and funding models that incentivize collaboration point to feasible, system-level changes that could unlock greater collective capacity without necessitating major new investments.

Our study findings align closely with recent calls for systems transformation in Canadian public health. The Chief Public Health Officer of Canada’s 2021 report emphasized the need for a more resilient, equitable and integrated public health system capable of addressing complex population health challenges through sustained investment, improved data infrastructure and stronger intersectoral partnerships.^30^ Similarly, the 2025 *Core Competencies for Public Health in Canada* reflects a modernized vision for public health practice that centres equity, complex problem solving, Indigenous engagement and digital capacity.^31^ These priorities echo the work of Mondal et al.^33^ who described organizational and leadership competencies such as systems thinking, strategic communication and the ability to adapt to complexities as foundational to public health capacity. Participants’ calls for long-term, coordinated CDP funding, equity-informed program design and infrastructure to support collaboration mirror these national and scholarly priorities.

Finally, while participants frequently emphasized pragmatic strategies such as modest, targeted investments or local partnerships to sustain CDP within current constraints, their accounts also reflected a recognition that these efforts alone are insufficient. For example, several quotes highlight a need for a long-promised but still absent shift in priorities from acute care to prevention and from short-term deliverables to long-term population health outcomes. Chronic underfunding and structural fragmentation were seen as systemic issues requiring incremental adjustments and broader transformations of how prevention is valued and governed in Canada.

In sum, this study highlights the urgency of addressing the structural and systemic barriers that continue to undermine CDP in Canada. Without long-term investment, policy coherence and equity-driven design, the gap between public health goals and organizational capacity will only widen. As governments pursue post-pandemic health system transformations, positioning CDP as a foundational—not peripheral—component of population health is critical.

***Strengths and limitations***

Study strengths include the use of a descriptive qualitative design, which enabled contextual insights into how barriers to CDP are experienced by practitioners with in-depth organizational knowledge. Thematic analysis was conducted independently by two researchers, enhancing analytical rigour and interpretive validity.^34^

Limitations include reliance on a single respondent per organization, which may have constrained the diversity of the perspectives captured. While the open-ended prompt was intentionally broad to encourage spontaneous reflection, it likely introduced variability in response depth and focus. Future research could incorporate follow-up interviews or targeted prompts to systematically explore specific thematic areas. 

All the participating organizations were well-established, with organizational age spanning from 25 to more than 100 years. Their perspectives likely reflect the experiences of mature organizations with greater resilience and the infrastructure to sustain CDP efforts despite chronic underfunding. Our findings may not fully capture the realities of newer or less-established organizations that ceased to exist between PHORCAST data collection waves. Future research should also examine how organizational age and maturity shape CDP capacity, especially under system stress.

***Policy recommendations***

Findings from this study support a more focused and evidence-anchored set of policy directions grounded in participants’ accounts. Rather than broad system reform, the participants emphasized the need for pragmatic actions to sustain CDP under current conditions. Five priority areas emerged. First, governments should provide stable, inflation-adjusted core funding for CDP to support staffing, program continuity and basic operational capacity.^29^,^35^ The participants consistently described flat or short-term funding as a central barrier that limits planning, scale-up and sustainability. 

Second, greater policy coherence is needed to reduce frequent shifts in priorities that compel organizations to continually redirect efforts in response to changing political agendas. The participants emphasized that prevention requires long time spans that are incompatible with short-term funding cycles. 

Third, intersectoral collaboration requires dedicated infrastructure.^32^,^36^ The participants highlighted the absence of intermediary supports, coordination mechanisms and partnership funding as barriers to sustained collaboration, despite a strong willingness to work across sectors. 

Fourth, CDP initiatives must be equity oriented and culturally responsive.^29^ The participants emphasized the need for approaches that extend beyond digital-only delivery models and that better engage Indigenous communities, older adults, rural populations and other groups for whom standard modalities are poorly aligned.^37^,^38^

Fifth, rebuilding CDP capacity following pandemic-related disruptions remains an urgent priority.^39^,^40^ The participants described lasting impacts on workforce stability, partnerships and institutional memory, indicating that recovery requires more than restarting programs.

## Conclusion

This study underscores that, two decades after the launch of PHORCAST, Canadian public health organizations continue to face persistent and, in some cases, intensified barriers. Without sustained investment, coherent policy direction and support for collaboration and equity responsive practice, CDP will remain vulnerable to ongoing and future system shocks.

## Acknowledgements

Katerina Maximova holds the Murphy Family Foundation Chair in Early Life Interventions. Maryam Marashi holds a doctoral fellowship from the Social Sciences and Humanities Research Council of Canada. Erin O’Loughlin held a post-doctoral salary award from the Fonds de recherche du Qubec – Sant (FRQ-S) during this study. Jennifer O’Loughlin held a Canada Research Chair in the Early Determinants of Adult Chronic Disease from 2004 to 2021.

## Funding

The PHORCAST project was supported by operational funds from the Canadian Institutes of Health Research (grant #170321).

## Conflicts of interest

Jennifer O’Loughlin is a member of the Editorial Board for this journal, but was not involved in the review process and editorial decision-making for this article. 

The authors have no competing interests.

## Authors’ contributions and statement

KM: Conceptualization, funding acquisition, methodology, project administration, supervision, writing—original draft, writing—review and editing.

MM: Formal analysis, writing—original draft, writing—review and editing. 

EKO’L: Formal analysis, writing—original draft, writing—review and editing. 

JO’L: Conceptualization, funding acquisition, methodology, writing—review and editing.

The content and views expressed in this article are those of the authors and do not necessarily reflect those of the Government of Canada.
